# Linking stomatal traits and expression of slow anion channel genes *HvSLAH1* and *HvSLAC1* with grain yield for increasing salinity tolerance in barley

**DOI:** 10.3389/fpls.2014.00634

**Published:** 2014-11-25

**Authors:** Xiaohui Liu, Michelle Mak, Mohammad Babla, Feifei Wang, Guang Chen, Filip Veljanoski, Gang Wang, Sergey Shabala, Meixue Zhou, Zhong-Hua Chen

**Affiliations:** ^1^School of Science and Health, University of Western SydneyPenrith, NSW, Australia; ^2^School of Chemical Engineering and Technology, Tianjin UniversityTianjin, China; ^3^School of Land and Food, University of TasmaniaHobart, TAS, Australia; ^4^College of Agriculture and Biotechnology, Zhejiang UniversityHangzhou, China; ^5^School of Environmental Science and Engineering, Tianjin UniversityTianjin, China

**Keywords:** soil salinity, stomata, gas exchange, quantitative trait loci, *HvSLAC1*, *HvSLAH1*, *Hordeum vulgare* L

## Abstract

Soil salinity is an environmental and agricultural problem in many parts of the world. One of the keys to breeding barley for adaptation to salinity lies in a better understanding of the genetic control of stomatal regulation. We have employed a range of physiological (stomata assay, gas exchange, phylogenetic analysis, QTL analysis), and molecular techniques (RT-PCR and qPCR) to investigate stomatal behavior and genotypic variation in barley cultivars and a genetic population in four experimental trials. A set of relatively efficient and reliable methods were developed for the characterization of stomatal behavior of a large number of varieties and genetic lines. Furthermore, we found a large genetic variation of gas exchange and stomatal traits in barley in response to salinity stress. Salt-tolerant cultivar CM72 showed significantly larger stomatal aperture under 200 mM NaCl treatment than that of salt-sensitive cultivar Gairdner. Stomatal traits such as aperture width/length were found to significantly correlate with grain yield under salt treatment. Phenotypic characterization and QTL analysis of a segregating double haploid population of the CM72/Gairdner resulted in the identification of significant stomatal traits-related QTLs for salt tolerance. Moreover, expression analysis of the slow anion channel genes *HvSLAH1* and *HvSLAC1* demonstrated that their up-regulation is linked to higher barley grain yield in the field.

## Introduction

Soil salinity is one of the most difficult challenges facing global agriculture as it endeavors to increase productivity to meet world crop demands for human consumption and animal fodder. A third of the world's agricultural land will be significantly affected by salinity by 2050. Low precipitation, high evaporation, irrigation with saline water, and poor agricultural practice are among the major contributors to increased soil salinity (Pitman and Lähli, 2002; Zhu, 2002; Munns et al., 2006; Munns and Tester, 2008). The levels of salinity in some agricultural areas have exceeded the threshold of 50% yield reduction of many commercial crops even salt-tolerant barley. Therefore, breeding crops with higher salt-tolerance is a current and serious concern in agriculture (Mano and Takeda, 1997; Munns and Tester, 2008; Xue et al., 2009; Shabala and Mackay, 2011).

Understanding how plants respond to salinity stress has resulted in an improvement in crop yields and is seen as one of the key strategies to deliver continued crop improvements through genetic engineering (Munns et al., 2006; Ullrich, 2011; Schroeder et al., 2013). Non-halophytic crop species are usually unable to withstand saline soils. Firstly, the presence of salts cause water in soil to be more tightly bound, therefore reducing the water availability to plants thus consequently causing dehydration in plants. Secondly, most plants are not able to avoid salt absorption into their root and leaf tissues. This leads to high salt accumulation, disrupting normal physiological and biochemical functions in plants (Chen et al., 2005; Munns et al., 2006; Shabala and Mackay, 2011; Ullrich, 2011). Barley (*Hordeum vulgare L*.) is the fourth largest cereal crop grown worldwide and has one major advantage over wheat and many other crops: that is its ability to tolerate higher levels of soil salinity (Munns et al., 2006; Ullrich, 2011). Although barley is relatively tolerant to salt, there are large variations between genotypes in their salinity tolerance. This led to the utilization of genetic diversity to meet the increasing need to understand the physiological and genetic responses of barley to salt stress (Zhu, 2002; Chen et al., 2007a,b; Munns and Tester, 2008; Xue et al., 2009; Ullrich, 2011; Zhou et al., 2012).

As a powerful tool to link phenotypic traits and genotypic markers for salinity tolerance, Quantitative Trait Loci (QTLs) analysis is feasible to fine-map some genes involved in particular major quantitative traits (Kearsey, 1998; Qiu et al., 2011). In the last two decades, QTLs associated with saline tolerance have been mapped in barley at the germination, seedling, and late growth stages by using various genetic populations (Mano and Takeda, 1997; Xue et al., 2009; Siahsar and Narouei, 2010; Qiu et al., 2011; Zhou, 2011). Breeding barley for adaptation to saline soil lies in a better understanding of molecular mechanisms involved in QTLs of stomatal distribution and opening and genes encoding membrane transporters. Stomata consist of specialized guard cells (two guard cells and two subsidiary cells for monocots), which regulate photosynthetic CO_2_ uptake and transpiration (Chen and Blatt, 2010; Kim et al., 2010; Chen et al., 2012; Hills et al., 2012). However, apart from correlating stomatal conductance to salinity stress, there is little systematic research for the identification of genetic control of stomatal behavior and its relationship to salinity tolerance in barley. Moreover, understanding the mechanisms of the complex network of regulatory genes of the control of stomata under salinity could be critical to reduce water loss and to maintain a high photosynthetic rate for better yield (Munns and Tester, 2008; Kim et al., 2010; Hedrich, 2012; Deinlein et al., 2014). Among the key regulatory genes, guard cell slow (or S-type) anion channels SLAC was found to be the “master switch” for stomatal closure (Vahisalu et al., 2008; Chen et al., 2010; Barbier-Brygoo et al., 2011; Geiger et al., 2011; Dreyer et al., 2012; Maierhofer et al., 2014; Zheng et al., 2014). The SLAC protein family comprised of SLAC1 and four SLAC1 homologs (SLAHs) in Arabidopsis (Negi et al., 2008; Vahisalu et al., 2008). The Arabidopsis genes *SLAH3* were found in mesophyll cells, but complementation of SLAC1 with SLAH3 recovered the anion channel function in guard cells. Therefore, it was proposed that SLAC1 and SLAH3 have an overlapping function (Negi et al., 2008; Geiger et al., 2011). Their difference is that SLAH3 predominately conducts nitrate but SLAC1 exhibited non-specific anion conductance. Given the key roles of SLAC and SLAH in stomatal closure, we propose that the *SLAC*/*SLAH* gene family might connect the stomatal response to salt stress with grain yield in barley.

The overarching hypothesis of this study was that physiological and molecular analysis of stomatal behavior contributes to the discovery of salt tolerance mechanisms in barley. The objectives of this study were to evaluate genetic variation of stomata behavior of barley under salinity stress and determine the links between physiological and molecular aspects of stomatal control and grain yield under saline condition in barley. It is likely that molecular markers and membrane transporter genes linked to stomatal traits could provide useful information for improving barley salinity tolerance in the future.

## Materials and methods

### Plant materials

Barley varieties and a double haploid (DH) population (CM72/Gairdner) were used for the four experimental trials. The DH population of 108 lines, developed by another culture of the F1 hybrid between CM72 (California Mariout 72, six-rowed; salt-tolerant), and Gairdner (an Australian malt barley cultivar, two-rowed; salt-sensitive) was used in Glasshouse Trial 2.

### Experimental trials

*Glasshouse Trial 1:* Seeds of 10 parental barley cultivars were sown and seedlings were thinned to 5 plants per pot with 4 replicates for both control and NaCl treatment. *Glasshouse Trial 2:* Seeds of 108 DH lines, their two parental cultivars (CM72 and Gairdner) and two reference cultivars (Yerong and Franklin) were sown and seedlings were thinned to 4 plants per pot with 4 replicates for both control and NaCl treatment. *Field Trial*: Fifty-one barley varieties from around the world were selected to evaluate their salinity tolerance in the field in Tasmania, Australia. Each variety was sown in six 1.5 m × 0.2 m plots with half of them being treated with NaCl 1 week after germination. Salt treatment were achieved by gradually adding salt to the three of the treatment plots at a rate of 500 g NaCl m^−2^ over 3 consecutive days. The final electrical conductivity (EC) of the salt treated soils was ~10 dS m^−1^. Normal pest and fertilizer application was employed. Leaf samples were collected for stomatal assay and gene expression analysis at Week 15. Plants were harvested for yield analysis at Week 20. *Glasshouse Trial 3:* The Field Trial was repeated in a glasshouse in New South Wales, Australia.

For the three Glasshouse Trials, plants were grown in two glasshouse rooms with identical conditions. All the plants were sown in 4-Litre pots containing potting mix and Osmocot® slow release fertilizer. Prior to salt treatment, all plants were watered and fed with full strength Hoagland's solution weekly. The glasshouse growth conditions were 24 ± 2°C and 60% relative humidity (RH) during the day, 22 ± 2°C and 70% RH at night. Broad spectrum growth lamps (600W HPS, GE Lighting, Smithfield, NSW, Australia) were used to provide a 12 h/12 h light/dark cycle. The average photosynthetically active radiation (PAR) received at the top of the plants was ~400 μmol m^−2^ s^−1^ over the duration of growth seasons. The plants were well watered to avoid drought stress during the experiments. Salt treatment was introduced 5 weeks after sowing at a concentration of 200 mM NaCl over 4 consecutive days at a rate of 50 mM (Munns et al., 2006; Chen et al., 2007c; Xue et al., 2009). All leached salt solutions were collected into the pot saucer and re-applied to ensure stability of NaCl concentrations across all treatment pots.

### Gas exchange measurement

Gas exchange was measurement as described in Chen et al. (2005); Mak et al. (2014); and O'carrigan et al. (2014). Net CO_2_ assimilation (*A*), intercellular CO_2_ concentration (*Ci*), stomata conductance (*g_s_*), transpiration rate (*T_r_*), leaf vapor pressure deficit (*VPD*), and leaf temperature (*T_leaf_*) measurements were collected from third fully expanded leaves 4 week after salt treatment using a LI-6400XT infrared gas analyser (Li-Cor Inc., Lincoln, NE, USA). The conditions in the measuring chamber were controlled at a flow rate of 500 mol s^−1^, a saturating PAR at 1500 μmol m^−2^ s^−1^, a CO_2_ level at 400 μmol mol^−1^ and a relative humidity of 65%. Gas exchange measurements were taken at the same time of day (approximately 10 a.m. to 4 p.m.) as for stomatal assay during full daylight for maximum photosynthesis. Each DH line was measured in its control and salt treatment pair to ensure comparative environmental and experimental conditions. Plants of each replication were randomly measured to minimize the effects of timing on gas exchange measurements.

### Stomatal assay

Stomatal imaging was conducted as described in Mak et al. (2014) and O'carrigan et al. (2014) with some modification. Third fully expanded leaves were collected from the glasshouse and transferred to the laboratory on tissue paper soaked in stomata stabilizing solution (50 mM KCl, 5 mM Na ± MES, pH 6.1) in petri dishes. The abaxial epidermal strips were then peeled and mounted on slides using a measuring solution (10 mM KCl, 5 mM Ca^2^ ± MES, pH 6.1). Prompt peeling and mounting was used as an important quality control step to ensure aperture images are true representations of the stomata found naturally on the whole plant in the glasshouse. Images of the stomata were taken using a CCD camera (NIS-F1 Nikon, Tokyo, Japan) attached to a microscope (Leica Microsystems AG, Solms, Germany). All images were managed using a Nikon NIS Element imaging software (Nikon, Tokyo, Japan) and measured with Image J software (NIH, USA). The 12 stomatal parameters were aperture length and width, aperture width/length, stomatal pore area, guard cell length, width and volume, subsidiary cell length, width and volume, and stomatal density and index.

### QTL analysis

The method for QTL analysis was essentially described in Xu et al. (2012). The DH population treated in 200 mM NaCl was used to identify QTLs controlling stomatal traits and gas exchange parameters using a map constructed with Diversity Array Technology (DArT) and Simple Sequence Repeat (SSR) markers. Using the software package MapQTL6.0 (Van Ooijen and Kyazma, 2009), QTLs were first analyzed by interval mapping (IM), followed by composite interval mapping (CIM). The closest marker at each putative QTL identified using interval mapping was selected as a cofactor and the selected markers were used as genetic background controls in the approximate multiple QTL model (MQM) of MapQTL6.0. Logarithm of the odds (LOD) threshold values for the presence of a QTL were estimated by performing the genome wide permutation tests implemented in MapQTL6.0 using at least 1000 permutations of the original data set for each trait, resulting in a 95% LOD threshold around 2.9. Two LOD support intervals around each QTL were established, by taking the two positions, left and right of the peak, that had LOD values of two less than the maximum (Van Ooijen and Kyazma, 2009), after performing restricted MQM mapping which does not use markers close to the QTL. The percentage of variance explained by each QTL (R^2^) was obtained using restricted MQM mapping implemented with MapQTL5.0. Graphical representation of linkage groups and QTL was carried out using MapChart 2.2 (Voorrips, 2002).

### Phylogenetic and alignment analysis

The phylogenetic tree of the SLAC/SLAH family from Arabidopsis and selected cereal crops was generated using MEGA 6 program (Tamura et al., 2013). The Maximum Likelihood with WAG+Freqs (F) and Gamma distribution (G) model was employed. Names of organisms and accession numbers are from the National Centre for Biological Information (NCBI). Statistical values of phylogeny were estimated by the bootstrap method and were shown at the nodes. Alignment of the barley slow anion channel like 1 (HvSLAH1) and slow anion channel 1 (HvSLAC1) with homologous proteins in selected species was conducted using the CLASTALW tool in the BioEditor software (http://www.mbio.ncsu.edu/bioedit/bioedit.html) with 1000-bootstraps.

### RT-PCR and qPCR

Flag leaves of 16 barley varieties were collected from the Field Trial and immediately frozen in liquid nitrogen. Total RNA was extracted with TRIZOL® reagent (Life Technologies, Mulgrave, VIC, Australia) following the manufacturer's instructions. One microgram of total RNA sample was used to synthesize cDNA with the sensiFAST™ Kit (Bioline, Alexandria, NSW, Australia). The concentration of isolated total RNA and cDNA was determined using a NanoDrop ND-1000™ spectrophotometer (Thermo Scientific, Waltham, MA, USA).

PCR of *HvSLAH1* and *HvSLAC1* in cDNA was performed as described in the GoTaq® Flexi DNA Polymerase protocol (Promega, Alexandria, NSW, Australia). PCR thermal cycling conditions were 2 min at 95°C for initial denaturation, followed by 35 cycles of denaturation at 95°C for 30 s, annealing at 60°C for 30 s and an extension at 72°C for 11 s, and a final extension at 72°C for 5 min. For each experiment, 500 ng of cDNA was used for PCR amplification in a 20-μl reaction. PCR products were separated on a 0.9% (w/v) agarose gel and visualized with GelRed (Biotium, USA). Gel images were processed with Image J software (NIH, USA) to estimate the integrated fluorescence intensity of each band similar to cell imaging analysis in Bonales-Alatorre et al. (2013).

Transcript levels of *HvSLAH1* and *HvSLAC1* in leaves of 16 barley varieties were also determined by quantitative Real Time PCR (qPCR) using gene specific primers (Table S1). Purified cDNA samples were assayed using a Rotor-Gene® Q6000 (QIAGEN, Hilden, Germany) with the SensiFAST™ SYBR No-ROX Kit (Bioline, Alexandria NSW, Australia). qPCR conditions were consisted of 3-step cycling: 1.) polymerase activation at 95°C for 2 min; 2.) 40 cycles were set up for 5 s denaturation at 95°C, 10 s annealing at 63°C, 15 s extension at 72°C; 3.) SYBR green signal data were acquired at end. *HvACTIN* was used as the reference gene for normalization, which were selected from a number of candidates (Cao et al., 2014). Data are averages of three independent biological experiments, where each has two technical replicates.

### Statistical analysis

Statistical significance between control and the treatments was examined using the Student's *t*-test at *P* < 0.05 and *P* < 0.01. Pearson correlation analysis of all the parameters was conducted using SPSS 20 (IBM, New York, USA) and SigmaPlot 12 (Systat Software Inc., San Jose, CA, USA).

## Results

### Large genetic diversity of salt tolerance in barley

**Figure 1:**
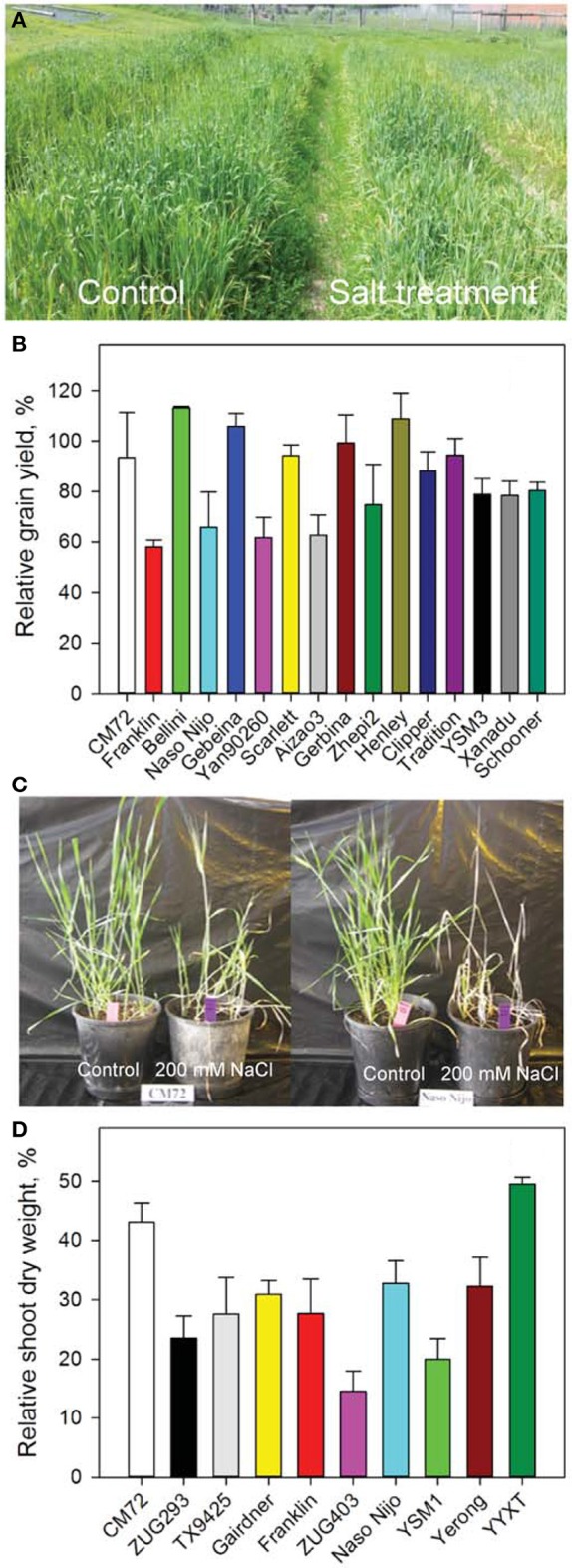
**Growth and yield of barley in the Field Trial and Glasshouse Trial 1**. **(A)** Image shows 15-week old barley plants grown in control (left) and 10 dS m^−1^ NaCl (right) in the Field Trial in Launceston, Tasmania. **(B)** Relative grain yields of 16 randomly selected genotypes. Data are percentage of grain yields under the salt treatment compared to control. Data are mean ± *SE* (*n* = 3). **(C)** Images of two representative varieties contrasting in their salinity tolerance. **(D)** Relative shoot dry weight of 10 varieties in Glasshouse Trial 1. Data shown are percentage of shoot dry weights under the salt treatment compared to control. Data are mean ± *SE* (*n* = 4).

Barley genotypes showed contrasting response to salinity stress (Figures 1A,B). Salt-tolerant variety CM72 gained a significantly higher (*P* < 0.05) grain yield than salt-sensitive Franklin (Figure 1B). Some of the European elite varieties such as Bellini and Henley even performed better under salinity stress than CM72. In the Glasshouse Trial 1, similar trend was observed where CM72 and YYXT retained significantly more shoot dry weight than those salt-sensitive genotypes (Figures 1C,D). A large genetic diversity thus provided a foundation for further analysis of gas exchange, stomatal traits, gene expression and grain yield in contrasting cultivars and among the DH lines.

### Gas exchange and stomatal traits differ significantly between salt-tolerant and sensitive genotypes

**Figure 2:**
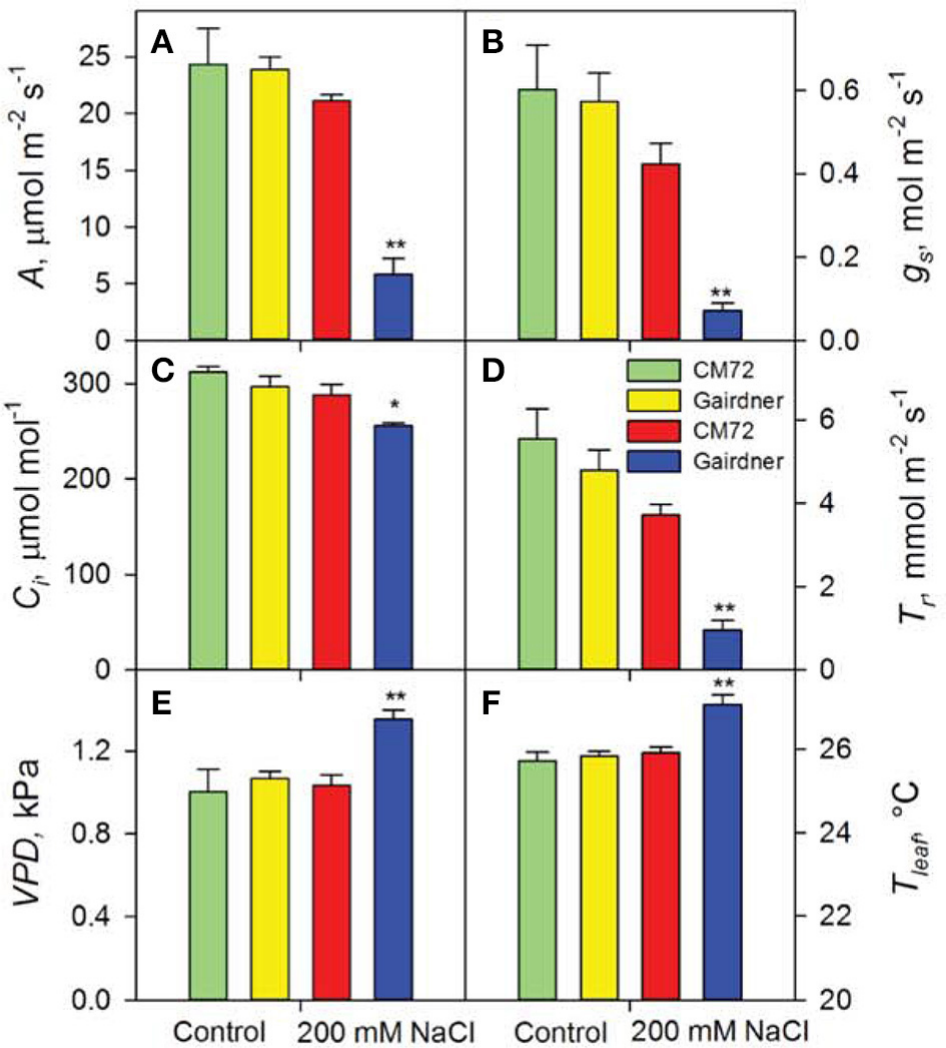
**Photosynthetic performance of two contrasting barley genotypes CM72 and Gairdner in the Glasshouse Trial 1**. Data show net CO_2_ assimilation **(A)**, stomatal conductance **(B)**, intracellular CO_2_ concentration **(C)**, transpiration rate **(D)**, leaf vapor pressure deficit **(E)**, and leaf temperature **(F)** in the Control (green and yellow bars) and in 200 mM NaCl (red and blue bars) for 4 weeks. Data are mean ± *SE* (*n* = 4). ^*^ and ^**^ indicate significant difference between CM72 and Gairdner under the salt treatment at *P* < 0.05 and *P* < 0.01, respectively.

**Figure 3:**
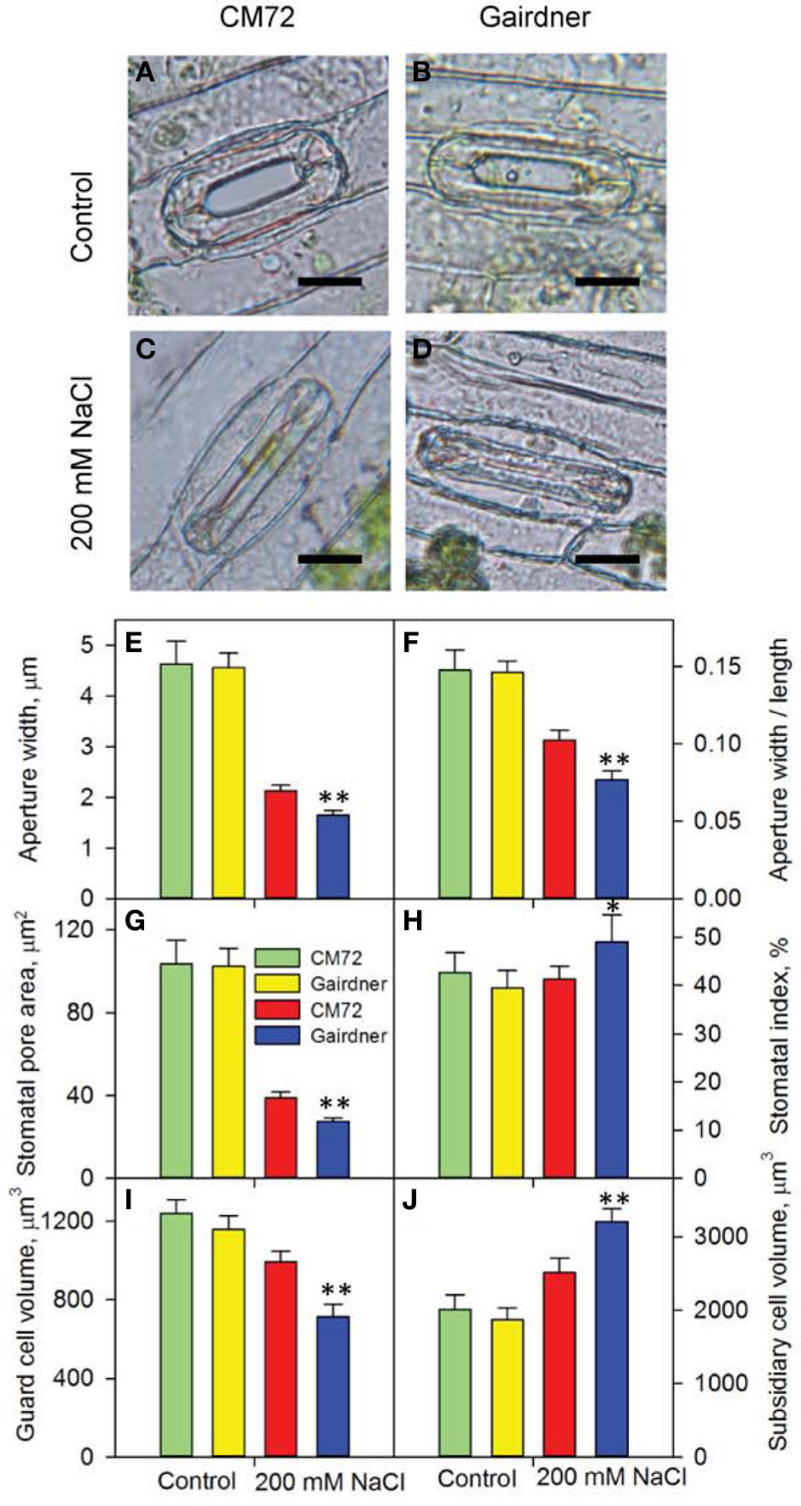
**Stomatal traits of two contrasting barley genotypes CM72 and Gairdner in the Glasshouse Trial 1**. **(A–D)** Representative images of single stomatal complex for control and 200 mM NaCl treatment. Scale bars = 10 μm. Data show aperture width **(E)**, aperture width/length **(F)**, stomatal pore area **(G)**, stomatal index **(H)**, guard cell volume **(I)** and subsidiary cell volume **(J)** in control (green and yellow bars) and in 200 mM NaCl (red and blue bars) for 4 weeks. Data are mean ± *SE* (*n* = 8–18 for stomatal index; *n* = 25–46 for rest of the stomatal traits). ^*^ and ^**^ indicate significant difference between CM72 and Gairdner in the salt treatment at *P* < 0.05 and *P* < 0.01, respectively.

Distinct performance between salt-tolerant CM72 and salt-sensitive Gairdner was observed for gas exchange parameters in both Glasshouse Trial 1 (Figure 2) and Glasshouse Trial 2 (data not shown). Four weeks of salt treatment at 200 mM NaCl imposed no obvious change in these parameters in CM72, however, Gairdner exhibited huge reductions (*P* < 0.01) in *A* (72.5%), *g_s_* (83.1%), and *T_r_* (74.4%) as well as significant increases (*P* < 0.01) in *VPD* (30.9%) and *T_leaf_* (4.4%) in comparison to these in CM72 (Figures 2A–F). We then further assessed the stomatal traits in these varieties in control (Figures 3A,B) and salinity treatment (Figures 3C,D) in Glasshouse Trial 1. There were negative impacts on the 11 stomatal traits 4 weeks after the imposition of salinity stress. Compared to Gairdner, CM72 gained 28.7, 32.9, 40.6, 39.6% larger aperture width, aperture width/length, stomatal pore area, guard cell volume and 19.1 and 27.4% lower stomatal index and subsidiary cell volume, respectively (Figures 3E–J). On the contrary, stomatal density was slightly increased (*P* > 0.05) by salinity treatment in both genotypes (Figure S1). Therefore, these stomatal traits showing large response to salt stress were employed for phenotypic characterization to identify potential salt tolerance-related QTLs in the CM72/Gairdner DH population.

### Stomatal traits contribute significantly to salinity tolerance and grain yield in barley

**Figure 4:**
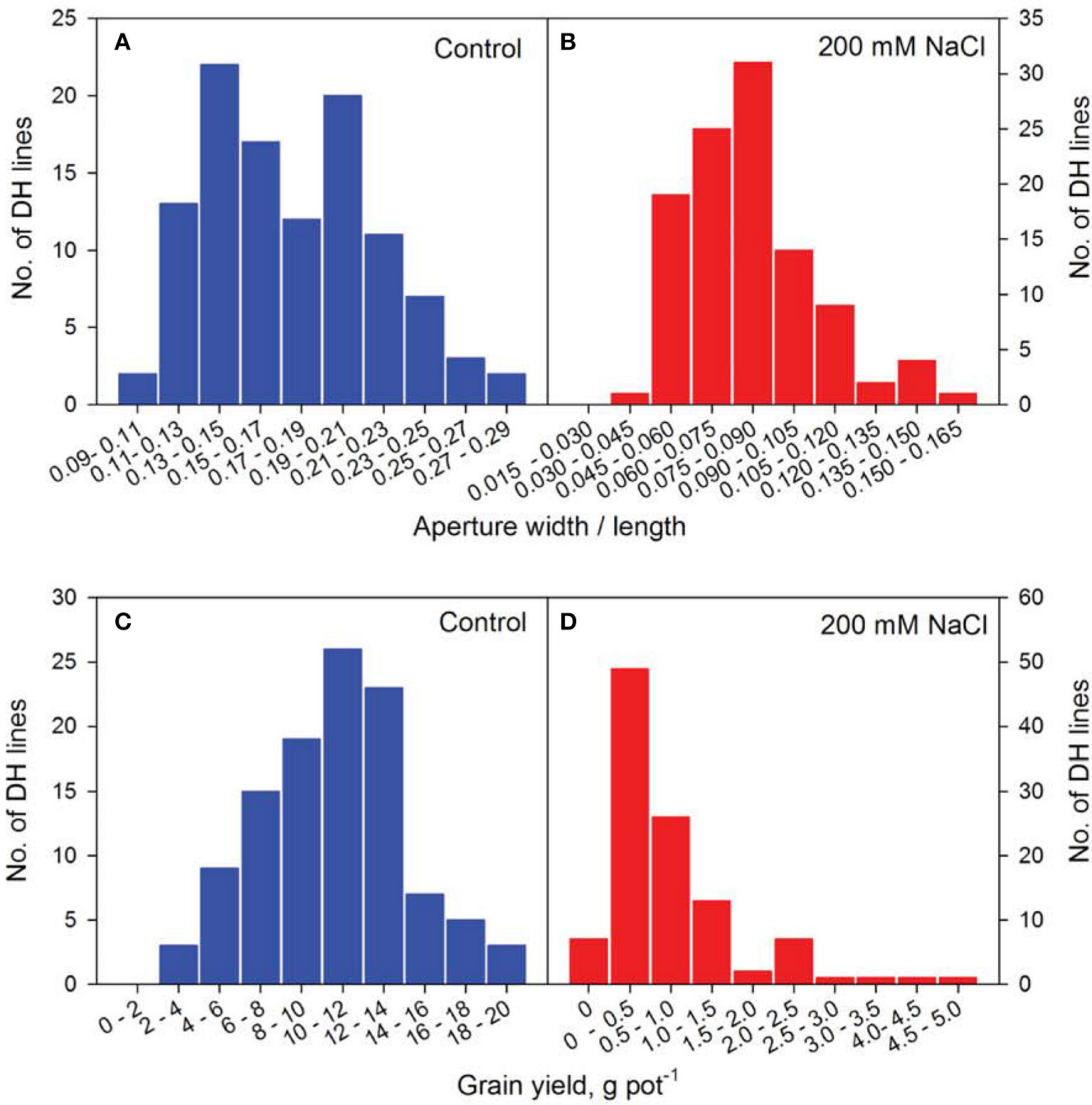
**Frequency distribution of representative phenotyping traits of the CM72/Gairdner DH population in Glasshouse Trial 2**. Frequency distribution of aperture width/length at control **(A)** and 200 mM NaCl **(B)** and grain yield at Control **(C)** and 200 mM NaCl **(D)** of the DH lines.

**Figure 5:**
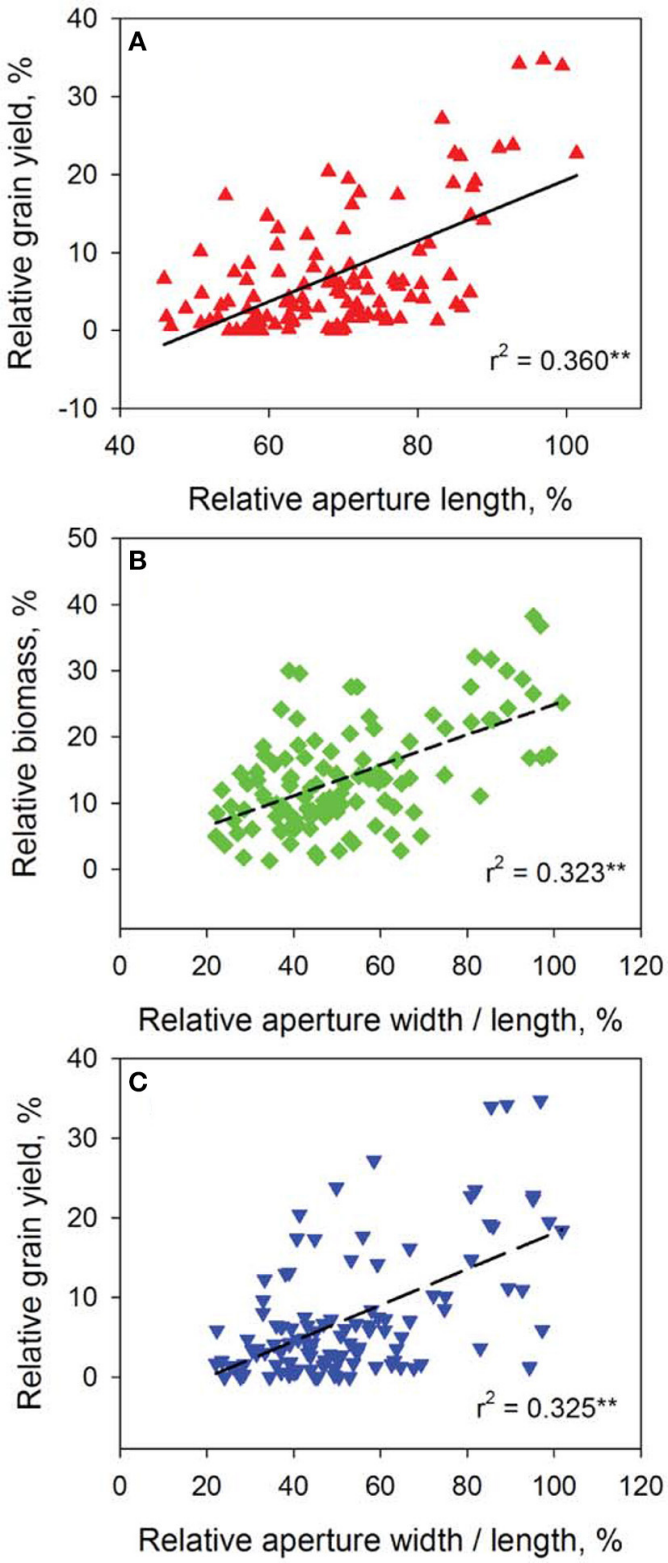
**Correlation analysis of representative stomatal and agronomical traits of the 108 DH lines**. Data show correlations between relative grain yield and relative aperture length **(A)**, relative aperture width/length and relative biomass **(B)** and relative grain yield **(C)**. ^**^P < 0.01.

**Figure 6:**
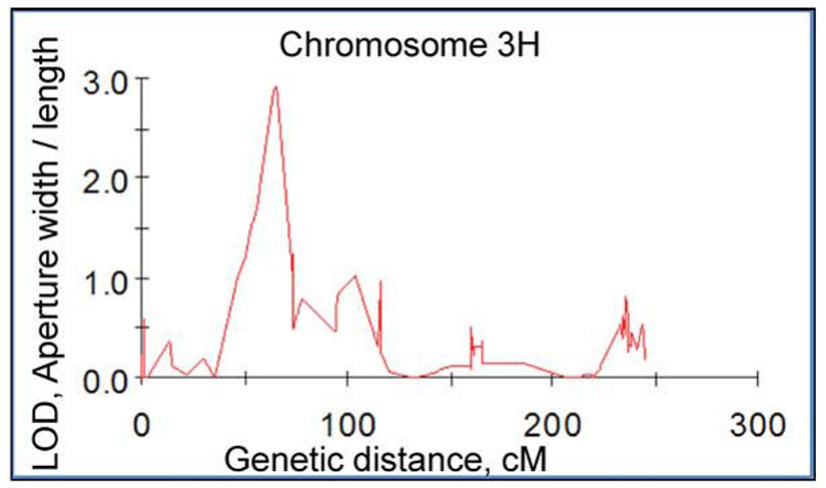
**A significant QTL for aperture width/length in salt tolerance**. This QTL was mapped on barley chromosome 3H in the double haploid population from CM72/Gairdner using a map constructed with Diversity Array Technology (DArT) and Simple Sequence Repeat (SSR) markers.

The speed and accuracy of measuring stomatal traits is a major obstacle for its use in breeding program. In this study, we have refined this technique by standardizing the measurements for stomatal traits (Figure 3). Our frequency distribution results highlighted a distinct pattern of change in aperture width/length and grain yield at control (Figures 4A,C) and 200 mM NaCl (Figures 4B,D) of the DH lines. Salinity stress has led both traits to skewed distributions toward the lower values (Figures 4B,D). The potential contribution of 12 stomatal and 6 gas exchange traits to salinity tolerance in barley were demonstrated by significant correlations between these traits and biomass and grain yield in Glasshouse Trial 2 (data not shown). For instance, relative aperture length and relative aperture width/length showed significant correlation with relative grain yield (Figures 5A,C). Additionally, relative aperture width/length was correlated with relative biomass with statistical significance at *P* < 0.01 (Figure 5B). Most of the stomatal traits in the DH lines showed distinct response to the salt treatment. The five best performing DH lines were 2.3, 3.3, and 2.2-fold higher in aperture width/length, stomatal area, and guard cell volume in contrast to the five least performing ones (Table S3). Interestingly, one QTL was identified for this important stomatal trait—aperture width/length. This QTL explained 11.8% of the phenotypic variation with an LOD value of 2.90 (Figure 6). The nearest marker for this QTL is bPb-4564. This QTL is located on a similar position to one of the QTLs for salinity tolerance based on leaf injury in 240 mM NaCl treatment (Fan et al. unpublished data).

### Slow anion channel genes positively regulate salt tolerance in barley

**Figure 7:**
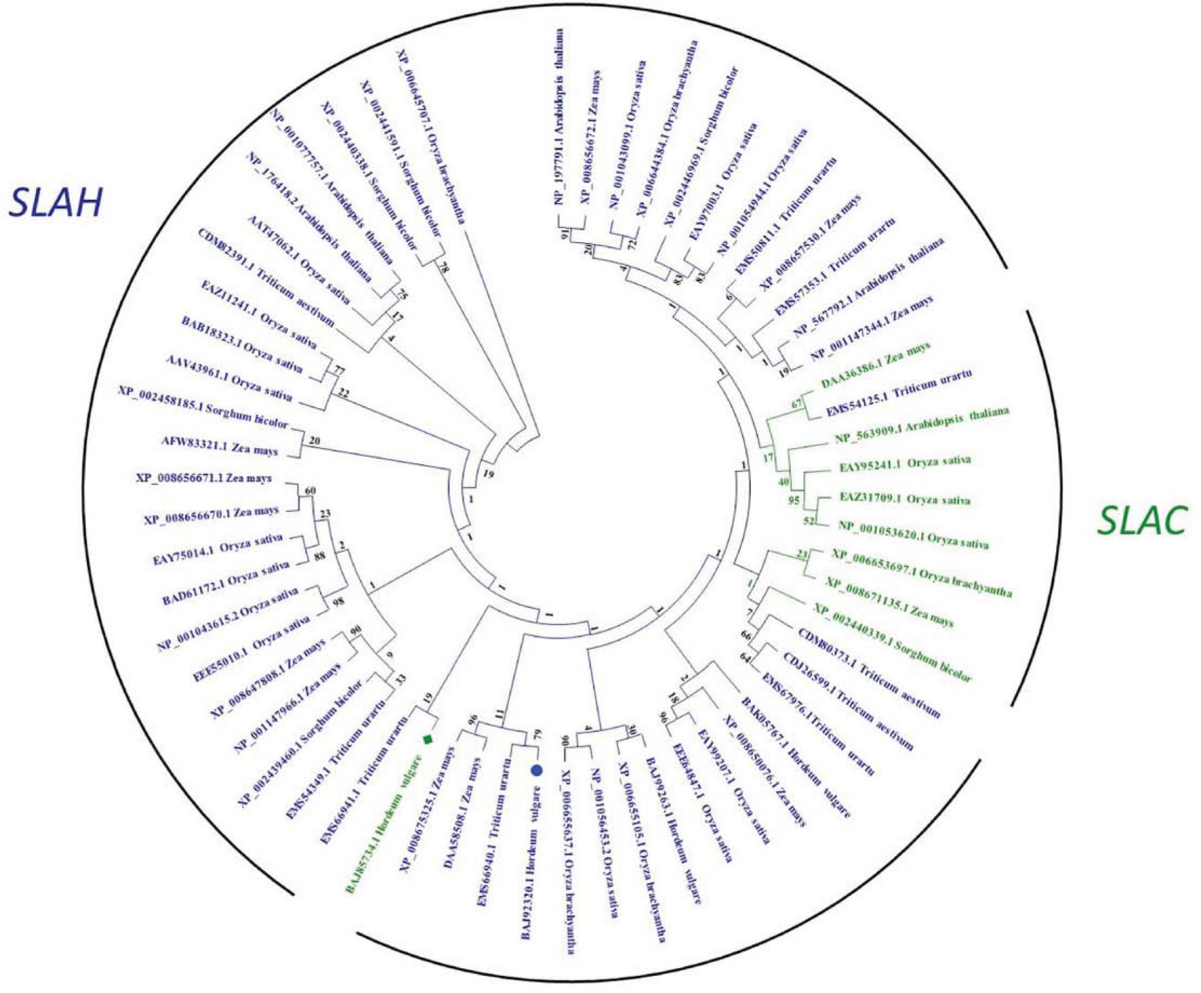
**Phylogenetic tree of SLAC and SLAH anion channel family in barley, Arabidopsis, and selected cereal crops**. Statistical values of phylogeny estimated by bootstrap method are shown at the nodes. HvSLAC1 and HvSLAH1 are marked by a green square and a blue circle, respectively.

Slow anion channels SLAC and SLAH are the key regulators of stomatal closure in plants (Vahisalu et al., 2008; Geiger et al., 2011; Dreyer et al., 2012; Hedrich, 2012). Our comparative genomic study indicated that SLACs and SLAHs exist in a large range of plant species (Figure 7). There are 4, 14, 10, 5, 3, and 3 SLAH-like genes in Arabidopsis, rice, maize, sorghum, wheat and barley, respectively. The numbers of corresponding SLAC-like genes are much lower (1, 3, 2, 1, 0, and 1) in these species (Figure 7, Figure S2). Further analysis showed that coding DNA sequence of *HvSLAC1* and *HvSLAH1* identified in this study have 55.0 and 54.8% homology to the well-characterized Arabidopsis *SLAC1* and *SLAH3*, respectively. Alignment analysis of amino acids revealed that HvSLAC1 and HvSLAH belong to SLAC1-group and SLAH1/4-group, respectively, as proposed in Dreyer et al. (2012). Their protein sequences showed a clear difference as compared to Arabidopsis and other cereal crops (Figure S2). These data provide a valuable source of information for future studies into the understanding of mechanisms of the different SLAC/SLAH contributions to barley salt tolerance.

**Figure 8:**
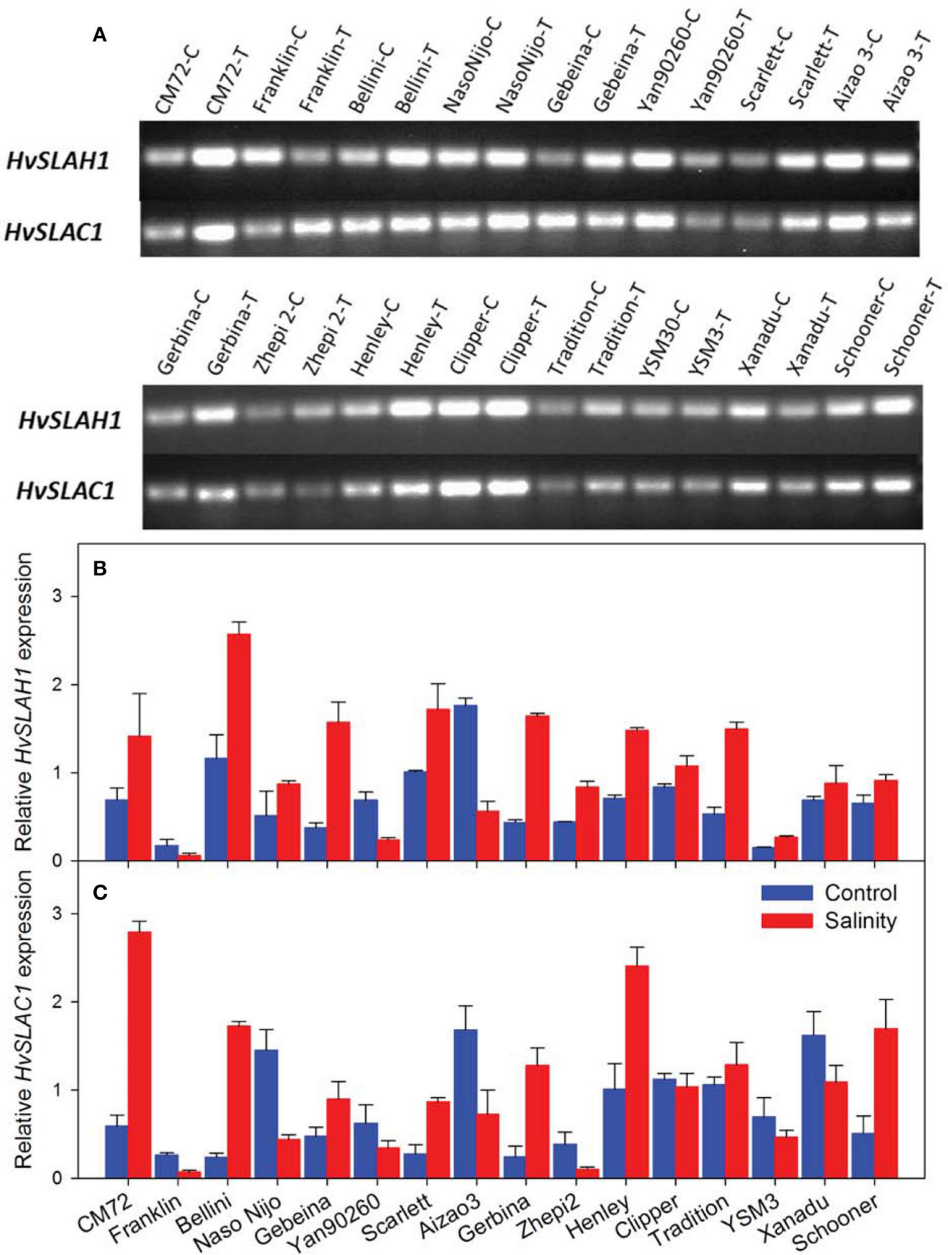
**Expression of *HvSLAH1* and *HvSLAC1* in leaves from the Field Trial**. **(A)** Real time-PCR gel electrophoresis of the two slow anion channel genes *HvSLAH1* and *HvSLAC1* in control **(C)** and salt treatment (T). Quantitative RT-PCR of the two slow anion channel genes *HvSLAH1* **(B)** and *HvSLAC1* **(C)** in the Control (blue bars) and salt treatment (red bars). *HvACTIN* was used as a reference gene. Data are average ± SD with three biological replicates.

**Figure 9:**
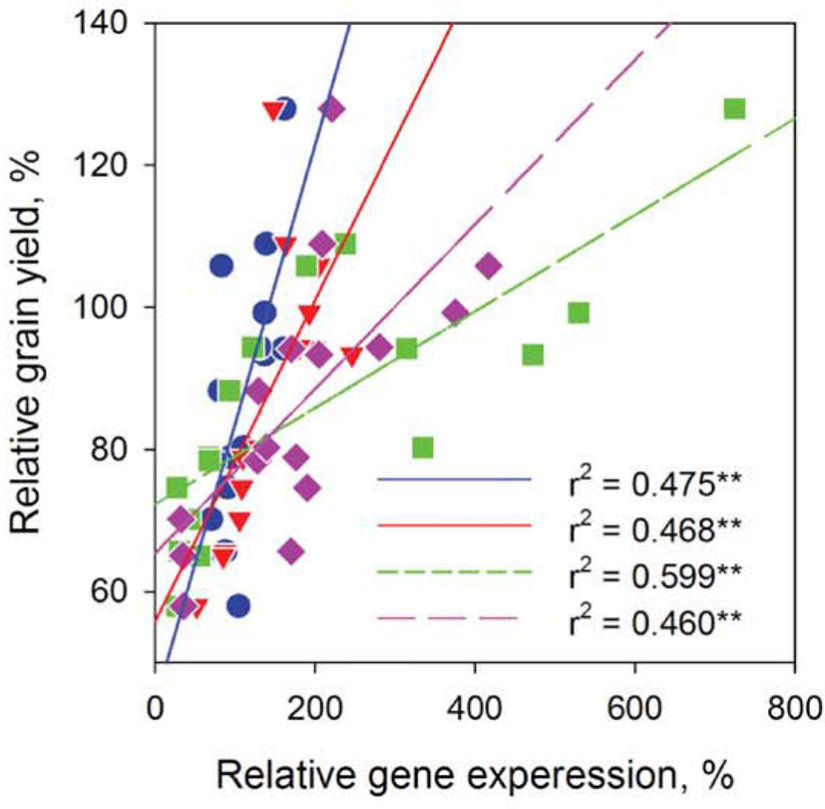
**Linking leaf *HvSLAH1* and *HvSLAC1* expression to barley grain yield in the Field Trial**. Relative gene expression was expressed as the transcripts under the salt treatment over that in control. Data were collected from 16 barley cultivars and fitted separately for each gene in both RT-PCR (blue circles and red triangles) and q-PCR (green squares and pink diamonds) experiments. ^**^P < 0.01.

We then conducted experiments to identify these barley *SLAC/SLAHs* in leaves from control and salt treatment in the field (Figures 1A, 8). As the first to report these genes in barley leaves, we named them as *HvSLAH1* and *HvSLAC1*. The expression of these two genes showed a large variation among the 16 genotypes in both control and salinity stress (Figure 8). Specifically, the salt-tolerant variety CM72 showed a NaCl-induced significant up-regulation of both *HvSLAH1* and *HvSLAC1*, which were significantly decreased or remained unchanged in salt-sensitive varieties Franklin and Naso Nijo (Figures 8B,C). Interestingly, the European varieties Bellini, Scarlett and Henley tended to have NaCl-induced up-regulation, but Asian varieties such as Naso Nijo, Yan90260, and Aizao3 showed down-regulation of *HvSLAH1* and *HvSLAC1* (Figure 8). Furthermore, highly significant (*P* < 0.01) positive correlations were found between the transcripts of two genes and salt tolerance of barley both in the field and the glasshouse (Figure 9 and Table S2). These were consistent across the RT-PCR gel integrated intensity based results and qPCR analysis (Figure 8). Additionally, the transcripts of the two genes from the samples collected in the Field Trial were even significantly linked to the visual salt tolerance score and grain yield in the Glasshouse Trial 3 (Table S2).

## Discussion

### Exploring the genetic diversity of barley for salt tolerance

The Hordeum genus, to which modern cultivated barley belongs, contains species which have adapted to grow in a wide variety of environmental conditions from the sub-arctic Scandinavia to the subtropical North Africa. Species include annual and perennial varieties with a wide array of shapes and structures, phenology and reproductive variations, suggesting that the genus is highly diverse and adaptable (Ullrich, 2011; Dai et al., 2012, 2014). It would not come as a surprise that landrace species of barley are found in saline meadows and marshes along coastal regions, utilizing morphologies like deep root system to evolve into salt-tolerant varieties such as *Hordeum marinum* (Ullrich, 2011). Recently, Tibetan wild barley (*Hordeum vulgare spp. spontaneum*), as one of the ancestors of cultivated barley (Dai et al., 2012, 2014), has been found to be even more tolerant to salinity (Qiu et al., 2011; Wu et al., 2011, 2013a,b). Furthermore, the level of salt tolerance in modern cultivated barley also differs significantly among genotypes. For example, seedlings of a barley cultivar California Mariout (CM) showed no growth retardation at 400 mM NaCl, while other barley genotypes were more affected (Epstein et al., 1980). Our results have further extended the large genetic variation to gas exchange (data not shown), stomatal traits (Figures 3–6) and the expression levels of *HvSLAH1* and *HvSLAC1* (Figure 8) in response to salinity stress in barley.

### Gas exchange traits may be accurate indicators for salinity tolerance but not for phenotyping study in barley

The effects of salinity stress on plants are largely due to osmotic stress and ion cytotoxicity (Zhu, 2002; Munns and Tester, 2008; Shabala and Mackay, 2011). Crop yield under salinity stress is a result of balancing the allocations of limited photosynthetic carbon gain toward both reproduction and growth. Photosynthesis is a multi-facetted process, which has dependencies on the availability of CO_2_, water and light (Wong et al., 1979; Farquhar and Sharkey, 1982). Upon the NaCl-induced stomatal closure, the reduced CO_2_ acquisition becomes the limiting factor in photosynthesis, resulting in leaf temperature increase, higher intracellular O_2_:CO_2_ ratio and oxidative stress (Farquhar and Sharkey, 1982; Chaves et al., 2009). For instance, salinity stress remarkably reduced *A* and *g_s_* in sorghum (Yan et al., 2012), wheat (Zheng et al., 2009) and barley (Jiang et al., 2006), the extent being considerably larger in salt-sensitive genotypes than salt-tolerant ones. Hence, the salt-tolerant genotypes could harmonize the relationship between CO_2_ assimilation (source) and the grain yield (sink) under the saline conditions (James et al., 2002). Measurement of *gs* provided the best information to assess genetic differences in barley for absolute performance when subjected to salinity stress (Jiang et al., 2006). In our experiments with gas exchange measurements (Glasshouse Trials 1, 2, and 3), some key traits (e.g., *g_s_*, *T_r_*, *VPD*) were found to be important indicators for salt tolerance after 4 weeks of 200 mM NaCl treatment. However, no significant QTLs were identified for the 6 gas exchange traits in the CM72/Gairdner DH population. We have reasoned that measuring these traits was highly variable over the course of the day and over a period up to 6 days. Also, many stomatal and non-stomatal limitations (James et al., 2002; Munns et al., 2006) are likely to make these measurements more difficult to control. In addition, there were limited replicates of gas exchange measurements as compared to up to 70 for the stomatal traits in some lines.

### Systematic investigation of stomatal traits for barley salinity tolerance

Most of the previously studies have mainly evaluated a small numbers of genotypes or a small genetic population for their stomatal behavior in response to salinity stress. Among those, limited traits such as stomatal length and width, stomatal density and index measured from leaf imprint have been used (Pallaghy, 1971; Edwards and Meidner, 1979; Aryavand et al., 2003; Mumm et al., 2011). Here, we have conducted a comprehensive comparative study with large datasets on stomatal traits (e.g., 75,640 data points for 12 parameters in Glasshouse Trial 2 alone). In addition, we have developed an efficient technique to obtain viable single-layer epidermal peels for microscopic recording of high resolution images (Figure S1, Figure 3). All of these have allowed the assessment of a large number of genotypes and relatively big genetic populations in the glasshouse and even in the field.

Salt-tolerant plant species *Aeluropus lagopoides* had fewer and smaller stomata on both leaf surfaces and stomata of *Osyris compressa* were found only on the abaxial surface under salinity stress (Naz et al., 2010). Salt tolerance was related to lower stomatal density and decreased stomatal area in *Sporobolus ioclados* (Naz et al., 2010) and *Chenopodium quinoa* (Shabala et al., 2013). Here, the correlation between stomatal density and salt tolerance in the barley DH population is marginal due to a lack of difference in the parental lines (Figure S1). Another study on this issue using 51 genotypes is currently under investigation. However, the relationships between aperture length, aperture width/length (Figure 5), guard cell and subsidiary cell volumes (data not shown) and grain yield in response to salinity stress and significant stomatal trait-related QTLs (Figure 6) have provided some insights into the contribution of stomatal traits in salinity tolerance in barley. The salt-tolerant varieties and best performing DH lines showed significantly larger stomatal pore area and guard cell volume as compared to the salt-sensitive and worst performing DH lines (Figures 3–6). Therefore, the reduction in photosynthesis and grain yield could be identified by stomatal assay to differentiate the responses among genotypes and genetic populations.

### Focus on membrane transporter regulation for future breeding for salt-tolerant crops

Significant progress has been made on the molecular mechanisms of membrane transport for salt tolerance in plants. Combining plant physiology, molecular breeding and genetic engineering have resulted in improvements in crop yields in saline soil (Munns and Tester, 2008; Tavakkoli et al., 2010; Schroeder et al., 2013; Deinlein et al., 2014). For instance, *TmHKT1;5-A* in the *Nax2* locus encodes a Na ± selective transporter located on the plasma membrane of root cells surrounding xylem vessels of bread wheat. It can remove Na^+^ from the xylem and reduce transport of Na^+^ to leaves. Field trials demonstrated that *TmHKT1;5-A* significantly reduces leaf Na^+^ and increases durum wheat grain yield by 25% (Munns et al., 2012). More recently, the expression of *AVP1*, an Arabidopsis gene encoding a vacuolar proton pumping pyrophosphatase, has been shown to improve the salinity tolerance of transgenic plants in both greenhouse and in field trials (Schilling et al., 2014). It is also the case for our comparative experimental trials in both field and glasshouse, where the grain yields and transcript of *HvSLAC1* and *HvSLAH1* are consistently and significantly correlated (Figure 9; Table S2).

Stomatal aperture in monocots is controlled by guard cells and their dynamic interactions and “shuttling” of ions and solutes with subsidiary cells. These processes are largely regulated by the concerted coordination of membrane transporters on both cell types (Raschke and Fellows, 1971; Edwards et al., 1976; Fairley-Grenot and Assmann, 1992; Philippar et al., 2003; Mumm et al., 2011). However, there are very few reports on guard cell transporters and salinity tolerance. Guard cell cation channels were found to be involved in Na ± induced stomatal closure in a halophyte *Aster tripolium*, which possesses no specific morphological adaptation to salinity. Short-term Na^+^ treatment (~30 min) induced stomatal opening. The plasma membrane K^+^ inward and outward rectifying channels were highly selective for K^+^ against Na^+^. Cytosolic Na^+^ accumulation over long-term has led to a delayed and dramatic deactivation of the K^+^ inward rectifying channels via an increase of cytosolic Ca^2+^ concentration (Very et al., 1998). In Arabidopsis, two SLAC/SLAH channels regulated anion efflux from the guard cells for stomatal closure and another two regulate anion/nitrate transport in roots (Vahisalu et al., 2008; Dreyer et al., 2012; Hedrich, 2012; Hills et al., 2012). Having higher transcripts of *HvSLAC1* and *HvSLAH1* or potentially higher number of these channels, salt-tolerant barley could have higher anion efflux for closure under salinity stress. However, the tolerant varieties showed larger stomatal aperture in salt treatment (Figure 3), which is seemingly contradicting to the up-regulation of *HvSLAC1* and *HvSLAH1*. Therefore, these genes could assist salt-tolerant varieties to have more rapid closure as an efficient tool to optimize water balance. On the other hand, one can argue that ion channels are closed most of the time and are not commonly considered as the limiting factor. Why do plants need to express more of *SLACs*/*SLAHs* if they can simply have available ones open for longer? Nevertheless, these controversies have provided new directions to investigate these genes in barley, Arabidopsis and other cereal crops using comparative genomic tool and the assembled barley genome (International Barley Genome Sequencing Consortium, 2012) in more depth in the future. It was also indicated that the other three HvSLAH channels might contribute to the regulation of stomata and nitrogen homeostasis. Further research is necessary to dissect the phylogenetic relationships and functional properties of the HvSLAHs and decipher their roles for salt tolerance in barley.

## Author contributions

Zhong-Hua Chen, and Meixue Zhou designed research; Xiaohui Liu, Michelle Mak, Mohammad Babla, Feifei Wang, and Filip Veljanoski performed research; Xiaohui Liu, Michelle Mak, Mohammad Babla, Guang Chen, Zhong-Hua Chen. analyzed data; Zhong-Hua Chen, Meixue Zhou, Gang Wang, Sergey Shabala, Xiaohui Liu, and Michelle Mak wrote the paper.

### Conflict of interest statement

Reviewer Guoping Zhang declares that, despite affiliational and co-author links with authors Guang Chen, Meixue Zhou and Sergey Shabala, the review process was handled objectively. The authors declare that the research was conducted in the absence of any commercial or financial relationships that could be construed as a potential conflict of interest.

## Supplementary material

Figure S1 **Stomatal density of two contrasting barley genotypes CM72 and Gairdner in the Glasshouse Trial 1**. **(A-D)** Representative stomatal images for control and 200 mM NaCl treatment. Scale bars = 40 μm. **(E)** Stomatal density in control (green and yellow bars) and in 200 mM NaCl (red and blue bars) for 4 weeks. Data are mean ± *SE* (*n* = 8–18).

Figure S2 **Alignment of HvSLAC1 and HvSLAH1 with homologous proteins in selected species**. **(A-C)** Alignment of HvSLAC1 and some SLACs from monocot plant species and Arabidopsis. **(D-G)** Alignment of HvSLAH1 and some S-type anion channel SLAH from monocot plant species and Arabidopsis. Identical residues among the other proteins are indicated with asterisks. Colons indicate amino acids with high similarity among the proteins. Dashes indicate gaps.

## References

[B1] Aryavand A. Ehdaie B. Tran B. Waines J. G. (2003). Stomatal frequency and size differentiate ploidy levels in *Aegilops neglecta*. Genet. Resour. Crop Evol. 50, 175–182 10.1023/A:1022941532372

[B2] Barbier-Brygoo H. De Angeli A. Filleur S. Frachisse J.-M. Gambale F. Thomine S.. (2011). Anion channels/transporters in plants: from molecular bases to regulatory networks. Annu. Rev. Plant Biol. 62, 25–51. 10.1146/annurev-arplant-042110-103741 21275645

[B3] Bonales-Alatorre E. Shabala S. Chen Z. H. Pottosin I. (2013). Reduced tonoplast fast-activating and slow-activating channel activity is essential for conferring salinity tolerance in a facultative halophyte, Quinoa. Plant Physiol. 162, 940–952. 10.1104/pp.113.216572 23624857 PMC3668082

[B4] Cao F. Sun H. Chen F. Wang F. Zhang G. Chen Z. (2014). Genome-wide transcriptome analysis reveals cadmium-induced differential expression of key genes in Cd-tolerant and -sensitive barley genotypes. BMC Genomics 15:611 10.1186/1471-2164-15-611 25038590 PMC4117959

[B5] Chaves M. Flexas J. Pinheiro C. (2009). Photosynthesis under drought and salt stress: regulation mechanisms from whole plant to cell. Ann. Bot. 103, 551–560. 10.1093/aob/mcn125 18662937 PMC2707345

[B6] Chen Z. Cuin T. A. Zhou M. Twomey A. Naidu B. P. Shabala S. (2007a). Compatible solute accumulation and stress-mitigating effects in barley genotypes contrasting in their salt tolerance. J. Exp. Bot. 58, 4245–4255. 10.1093/jxb/erm284 18182428

[B7] Chen Z.-H. Blatt M. R. (2010). Membrane transport in guard cells, in Encyclopedia of Life Sciences (Chichester: John Wiley & Sons, Ltd.), 1–13 10.1002/9780470015902.a0021630

[B8] Chen Z. H. Hills A. Batz U. Amtmann A. Lew V. L. Blatt M. R. (2012). Systems dynamic modeling of the stomatal guard cell predicts emergent behaviors in transport, signaling, and volume control. Plant Physiol. 159, 1235–1251. 10.1104/pp.112.197350 22635112 PMC3404696

[B9] Chen Z.-H. Hills A. Lim C. K. Blatt M. R. (2010). Dynamic regulation of guard cell anion channels by cytosolic free Ca^2+^ concentration and protein phosphorylation. Plant J. 61, 816–825. 10.1111/j.1365-313X.2009.04108.x 20015065

[B10] Chen Z. Newman I. Zhou M. Mendham N. Zhang G. Shabala S. (2005). Screening plants for salt tolerance by measuring K^+^ flux: a case study for barley. Plant Cell Environ. 28, 1230–1246 10.1111/j.1365-3040.2005.01364.x

[B11] Chen Z. Pottosin I. I. Cuin T. A. Fuglsang A. T. Tester M. Jha D.. (2007b). Root plasma membrane transporters controlling K^+^/Na^+^ homeostasis in salt stressed barley. Plant Physiol. 145, 1714–1725. 10.1104/pp.107.110262 17965172 PMC2151677

[B12] Chen Z. Zhou M. Newman I. A. Mendham N. J. Zhang G. Shabala S. (2007c). Potassium and sodium relations in salinised barley tissues as a basis of differential salt tolerance. Funct. Plant Biol. 34, 150–162 10.1071/FP06237 32689341

[B13] Dai F. Chen Z.-H. Li Z. Wang X. Cai S. Wu D.. (2014). Transcriptome profiling reveals mosaic genomic origins of modern cultivated barley. Proc. Natl. Acad. Sci. U.S.A. 111, 13403–13408. 10.1073/pnas.1414335111 25197090 PMC4169977

[B14] Dai F. Nevo E. Wu D. Comadran J. Zhou M. Qiu L.. (2012). Tibet is one of the centers of domestication of cultivated barley. Proc. Natl. Acad. Sci. U.S.A. 109, 16969–16973. 10.1073/pnas.1215265109 23033493 PMC3479512

[B15] Deinlein U. Stephan A. B. Horie T. Luo W. Xu G. Schroeder J. I. (2014). Plant salt-tolerance mechanisms. Trends Plant Sci. 19, 371–379. 10.1016/j.tplants.2014.02.001 24630845 PMC4041829

[B16] Dreyer I. Gomez-Porras J. L. Riaño-Pachón D. M. Hedrich R. Geiger D. (2012). Molecular evolution of slow and quick anion channels (SLACs and QUACs/ALMTs). Front. Plant Sci. 3:263. 10.3389/fpls.2012.00263 23226151 PMC3509319

[B17] Edwards M. Meidner H. (1979). Direct measurements of turgor pressure potentialsIV. Naturally occurring pressures in guard cells and their relation to solute and matric potentials in the epidermis. J. Exp. Bot. 30, 829–837 10.1093/jxb/30.4.829

[B18] Edwards M. Meidner H. Sheriff D. W. (1976). Direct measurements of turgor pressure potentials of guard cells II. The mechanical advantage of subsidiary cells, the spannunqsphase, and the optimum leaf water deficit. J. Exp. Bot. 27, 163–171 10.1093/jxb/27.1.163

[B19] Epstein E. Norlyn J. D. Rush D. W. Kingsbur Y. R. W. Kelley D. B. Cunningham G. A.. (1980). Saline culture of crops: a genetic approach. Science 210, 399–404. 10.1126/science.210.4468.399 17837407

[B20] Fairley-Grenot K. A. Assmann S. M. (1992). Whole-cell K^+^ current across the plasma membrane of guard cells from a grass: *Zea mays*. Planta 186, 282–293. 10.1007/BF00196258 24186668

[B21] Farquhar G. Sharkey T. (1982). Stomatal conductance and photosynthesis. Annu. Rev. Plant Physiol. 33, 317–345 10.1146/annurev.pp.33.060182.001533

[B22] Geiger D. Maierhofer T. Al-Rasheid K. A. Scherzer S. Mumm P. Liese A.. (2011). Stomatal closure by fast abscisic acid signaling is mediated by the guard cell anion channel SLAH3 and the receptor RCAR1. Sci. Signal. 4, ra32. 10.1126/scisignal.2001346 21586729

[B23] Hedrich R. (2012). Ion channels in plants. Physiol. Rev. 92, 1777–1811. 10.1152/physrev.00038.2011 23073631

[B24] Hills A. Chen Z. H. Amtmann A. Blatt M. R. Lew V. L. (2012). OnGuard, a computational platform for quantitative kinetic modeling of guard cell physiology. Plant Physiol. 159, 1026–1042. 10.1104/pp.112.197244 22635116 PMC3387691

[B25] International Barley Genome Sequencing Consortium. (2012). A physical, genetic and functional sequence assembly of the barley genome. Nature 491, 711–716. 10.1038/nature11543 23075845

[B26] James R. A. Rivelli A. R. Munns R. Von Caemmerer S. (2002). Factors affecting CO_2_ assimilation, leaf injury and growth in salt-stressed durum wheat. Funct. Plant Biol. 29, 1393–1403 10.1071/FP02069 32688739

[B27] Jiang Q. Roche D. Monaco T. Durham S. (2006). Gas exchange, chlorophyll fluorescence parameters and carbon isotope discrimination of 14 barley genetic lines in response to salinity. Field Crops Res. 96, 269–278 10.1016/j.fcr.2005.07.010

[B28] Kearsey M. (1998). The principles of QTL analysis (a minimal mathematics approach). J. Exp. Bot. 49, 1619–1623 10.1093/jxb/49.327.1619

[B29] Kim T. H. Böhmer M. Hu H. Nishimura N. Schroeder J. I. (2010). Guard cell signal transduction network: advances in understanding abscisic acid, CO_2_, and Ca^2+^ signaling *Annu*. Rev. Plant Biol. 61, 561–591. 10.1146/annurev-arplant-042809-112226 20192751 PMC3056615

[B30] Maierhofer T. Lind C. Hüttl S. Scherzer S. Papenfuß M. Simon J.. (2014). A single-pore residue renders the Arabidopsis root anion channel SLAH2 highly nitrate selective. Plant Cell 26, 2554–2567. 10.1105/tpc.114.125849 24938289 PMC4114951

[B31] Mak M. Babla M. Xu S.-C. O'carrigan A. Liu X.-H. Gong Y.-M. (2014). Leaf mesophyll K^+^, H^+^ and Ca^2+^ fluxes are involved in drought-induced decrease in photosynthesis and stomatal closure in soybean. Environ. Exp. Bot. 98, 1–12 10.1016/j.envexpbot.2013.10.003

[B32] Mano Y. Takeda K. (1997). Mapping quantitative trait loci for salt tolerance at germination and the seedling stage in barley (*Hordeum vulgare* L.). Euphytica 94, 263–272 10.1023/A:1002968207362

[B33] Mumm P. Wolf T. Fromm J. Roelfsema M. R. G. Marten I. (2011). Cell type-specific regulation of ion channels within the maize stomatal complex. Plant Cell Physiol. 52, 1365–1375. 10.1093/pcp/pcr082 21690176

[B34] Munns R. James R. A. Läuchli A. (2006). Approaches to increasing the salt tolerance of wheat and other cereals. J. Exp. Bot. 57, 1025–1043. 10.1093/jxb/erj100 16510517

[B35] Munns R. James R. A. Xu B. Athman A. Conn S. J. Jordans C.. (2012). Wheat grain yield on saline soils is improved by an ancestral Na^+^ transporter gene. Nat. Biotechnol. 30, 360–364. 10.1038/nbt.2120 22407351

[B36] Munns R. Tester M. (2008). Mechanisms of salinity tolerance. Annu. Rev. Plant Biol. 59, 651–681. 10.1146/annurev.arplant.59.032607.092911 18444910

[B37] Naz N. Hameed M. Ashraf M. Al-Qurainy F. Arshad M. (2010). Relationships between gas-exchange characteristics and stomatal structural modifications in some desert grasses under high salinity. Photosynthetica 48, 446–456 10.1007/s11099-010-0059-7

[B38] Negi J. Matsuda O. Nagasawa T. Oba Y. Takahashi H. Kawai-Yamada M.. (2008). CO_2_ regulator SLAC1 and its homologues are essential for anion homeostasis in plant cells. Nature 452, 483–486. 10.1038/nature06720 18305482

[B39] O'carrigan A. Hinde E. Lu N. Xu X.-Q. Duan H. Huang G. (2014). Effects of light irradiance on stomatal regulation and growth of tomato. Environ. Exp. Bot. 98, 65–73 10.1016/j.envexpbot.2013.10.007

[B40] Pallaghy C. K. (1971). Stomatal movement and potassium transport in epidermal strips of *Zea mays*. Planta 101, 287–295. 10.1007/BF00398115 24488473

[B41] Philippar K. Buchsenschutz K. Abshagen M. Fuchs I. Geiger D. Lacombe B.. (2003). The K^+^ channel KZM1 mediates potassium uptake into the phloem and guard cells of the C4 grass *Zea mays*. J. Biol. Chem. 278, 16973–16981. 10.1074/jbc.M212720200 12611901

[B42] Pitman M. G. Lähli A. (2002). Global impact of salinity and agricultural ecosystems, in Salinity: Environment-Plants-Molecules, eds Läuchli A. Lüttge U. (Dordrecht:Springer), 3–20.

[B43] Qiu L. Wu D. Ali S. Cai S. Dai F. Jin X.. (2011). Evaluation of salinity tolerance and analysis of allelic function of *HvHKT1* and *HvHKT2* in Tibetan wild barley. Theor. Appl. Genet. 122, 695–703. 10.1007/s00122-010-1479-2 20981400

[B44] Raschke K. Fellows M. P. (1971). Stomatal movement in *Zea mays* shuttle of potassium and chloride between guard cells and subsidiary cells. Planta 101, 296–316. 10.1007/BF00398116 24488474

[B45] Schilling R. K. Marschner P. Shavrukov Y. Berger B. Tester M. Roy S. J.. (2014). Expression of the Arabidopsis vacuolar H^+^-pyrophosphatase gene (AVP1) improves the shoot biomass of transgenic barley and increases grain yield in a saline field. Plant Biotechnol. J. 12, 378–386. 10.1111/pbi.12145 24261956

[B46] Schroeder J. I. Delhaize E. Frommer W. B. Guerinot M. L. Harrison M. J. Herrera-Estrella L.. (2013). Using membrane transporters to improve crops for sustainable food production. Nature 497, 60–66. 10.1038/nature11909 23636397 PMC3954111

[B47] Shabala S. Hariadi Y. Jacobsen S.-E. (2013). Genotypic difference in salinity tolerance in quinoa is determined by differential control of xylem Na^+^ loading and stomatal density. J. Plant Physiol. 170, 906–914. 10.1016/j.jplph.2013.01.014 23485259

[B48] Shabala S. Mackay A. (2011). Ion transport in halophytes. Adv. Bot. Res. 57, 151–199 10.1016/B978-0-12-387692-8.00005-9

[B49] Siahsar B. Narouei M. (2010). Mapping QTLs of physiological traits associated with salt tolerance in ‘Steptoe’×‘Morex’ doubled haploid lines of barley at seedling stage. J. Food Agric. Environ. 8, 751–759.

[B50] Tamura K. Stecher G. Peterson D. Filipski A. Kumar S. (2013). MEGA6: molecular evolutionary genetics analysis version 6.0. Mol. Biol. Evol. 30, 2725–2729. 10.1093/molbev/mst197 24132122 PMC3840312

[B51] Tavakkoli E. Rengasamy P. McDonald G. K. (2010). The response of barley to salinity stress differs between hydroponic and soil systems. Funct. Plant Biol 37, 621–633 10.1071/FP09202

[B52] Ullrich S. E. (2011). Barley: Production, Improvement and Uses. West Sussex: Wiley Blackwell.

[B53] Vahisalu T. Kollist H. Wang Y.-F. Nishimura N. Chan W.-Y. Valerio G.. (2008). SLAC1 is required for plant guard cell S-type anion channel function in stomatal signalling. Nature 452, 487–491. 10.1038/nature06608 18305484 PMC2858982

[B54] Van Ooijen J. W. Kyazma B. V. (2009). “MapQTL 6” Software for the Mapping of Quantitative Trait Loci in Experimental Populations of Diploid Species. Kyazma BV: Wageningen.

[B55] Very A. A. Robinson M. F. Mansfield T. A. Sanders D. (1998). Guard cell cation channels are involved in Na^+^-induced stomatal closure in a halophyte. Plant J. 14, 509–521 10.1046/j.1365-313X.1998.00147.x

[B56] Voorrips R. E. (2002). MapChart: software for the graphical presentation of linkage maps and QTLs. J. Hered. 93, 77–78. 10.1093/jhered/93.1.77 12011185

[B57] Wong S. C. Cowan I. R. Farquhar G. D. (1979). Stomatal conductance correlates with photosynthetic capacity. Nature 282, 424–426 10.1038/282424a0

[B58] Wu D. Cai S. Chen M. Ye L. Zhang H. Dai F.. (2013a). Tissue metabolic responses to salt stress in wild and cultivated barley. PLoS ONE 8:e55431. 10.1371/journal.pone.0055431 23383190 PMC3561194

[B59] Wu D. Qiu L. Xu L. Ye L. Chen M. Sun D.. (2011). Genetic variation of *HvCBF* genes and their association with salinity tolerance in Tibetan annual wild barley. PLoS ONE 6:e22938 10.1371/journal.pone.0022938 21829562 PMC3145780

[B60] Wu D. Shen Q. Cai S. Chen Z.-H. Dai F. Zhang G. (2013b). Ionomic responses and correlations between elements and metabolites under salt stress in wild and cultivated barley. Plant Cell Physiol. 54, 1976–1988. 10.1093/pcp/pct134 24058150

[B61] Xu R. Wang J. Li C. Johnson P. Lu C. Zhou M. (2012). A single locus is responsible for salinity tolerance in a Chinese landrace barley (*Hordeum vulgare* L.). PLoS ONE 7:e43079. 10.1371/journal.pone.0043079 22916210 PMC3423432

[B62] Xue D. Huang Y. Zhang X. Wei K. Westcott S. Li C. (2009). Identification of QTLs associated with salinity tolerance at late growth stage in barley. Euphytica 169, 187–196 10.1007/s10681-009-9919-2

[B63] Yan K. Chen P. Shao H. Zhao S. Zhang L. Zhang L. (2012). Responses of photosynthesis and photosystem II to higher temperature and salt stress in *Sorghum*. J. Agron. Crop Sci. 198, 218–226 10.1111/j.1439-037X.2011.00498.x

[B64] Zheng X. He K. Kleist T. Chen F. Luan S. (2014). Anion channel SLAH3 functions in nitrate−dependent alleviation of ammonium toxicity in Arabidopsis. Plant Cell Environ. 10.1111/pce.12389 24944085

[B65] Zheng Y. H. Xu X. B. Wang M. Y. Zheng X. H. Li Z. J. Jiang G. M. (2009). Responses of salt-tolerant and intolerant wheat genotypes to sodium chloride Photosynthesis, antioxidants activities, and yield. Photosynthetica 47, 87–94 10.1007/s11099-009-0014-7

[B66] Zhou G. Johnson P. Ryan P. R. Delhaize E. Zhou M. (2012). Quantitative trait loci for salinity tolerance in barley (*Hordeum vulgare* L.). Mol. Breed. 29, 427–436. 10.1007/s11032-011-9559-922916210

[B67] Zhou M. (2011). Accurate phenotyping reveals better QTL for waterlogging tolerance in barley. Plant Breed. 130, 203–208 10.1111/j.1439-0523.2010.01792.x

[B68] Zhu J.-K. (2002). Salt and drought stress signal transduction in plants. Annu. Rev. Plant Biol. 53, 247–267. 10.1146/annurev.arplant.53.091401.143329 12221975 PMC3128348

