# Supplementary figures and images for: Linking stomatal traits and expression of slow anion channel genes HvSLAH1 and HvSLAC1 with grain yield for increasing salinity tolerance in barley
Source: Front Plant Sci. 2014 Nov 25;5:634. doi: 10.3389/fpls.2014.00634 (PMC4243495; doi:10.3389/fpls.2014.00634)

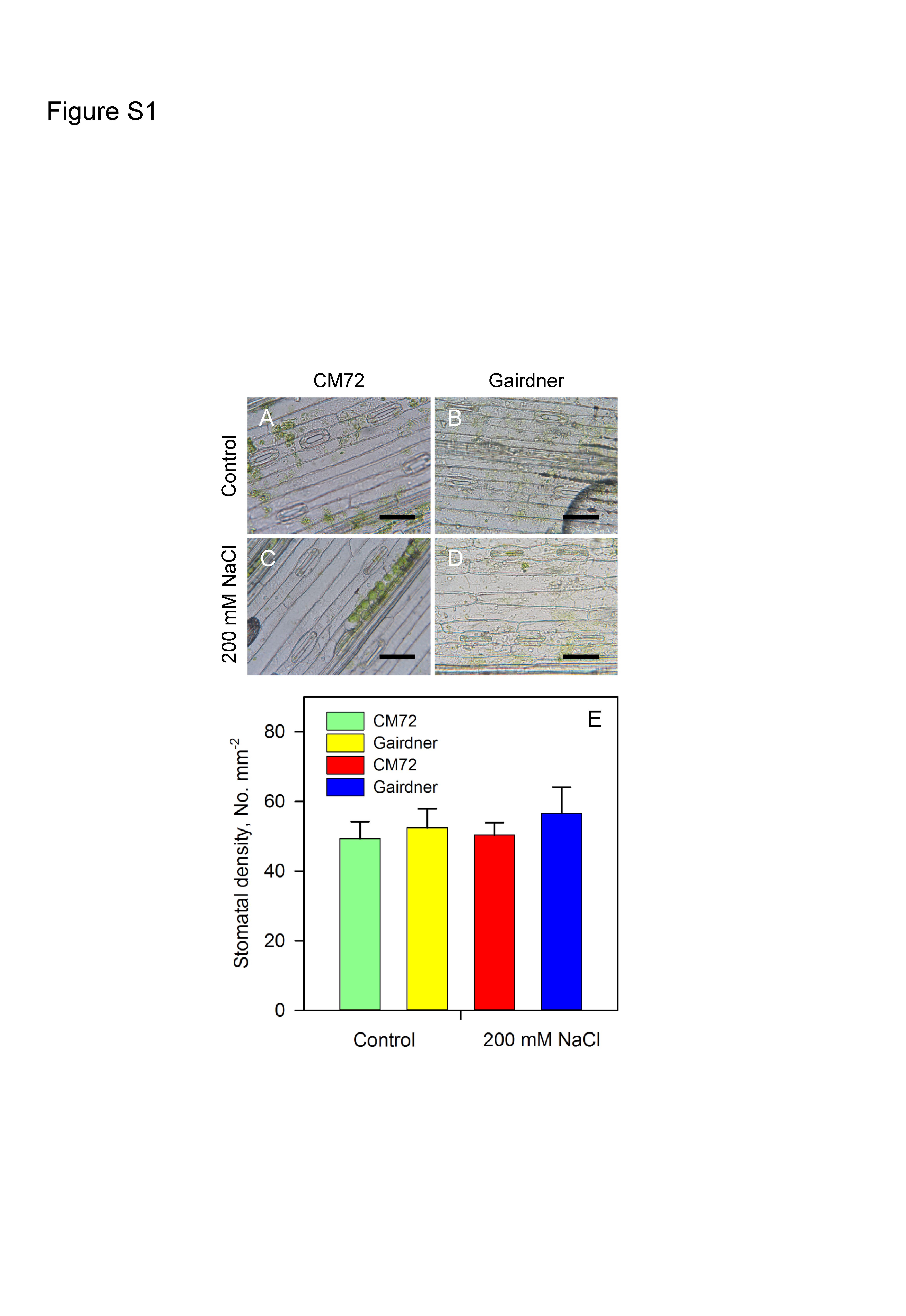

Supplement: Figure S1 — Stomatal density of two contrasting barley genotypes CM72 and Gairdner in the Glasshouse Trial 1. (A-D) Representative stomatal images for control and 200 mM NaCl treatment. Scale bars = 40 μm. (E) Stomatal density in control (green and yellow bars) and in 200 mM NaCl (red and blue bars) for 4 weeks. Data are mean ± SE (n = 8–18). [file Image1.JPEG]

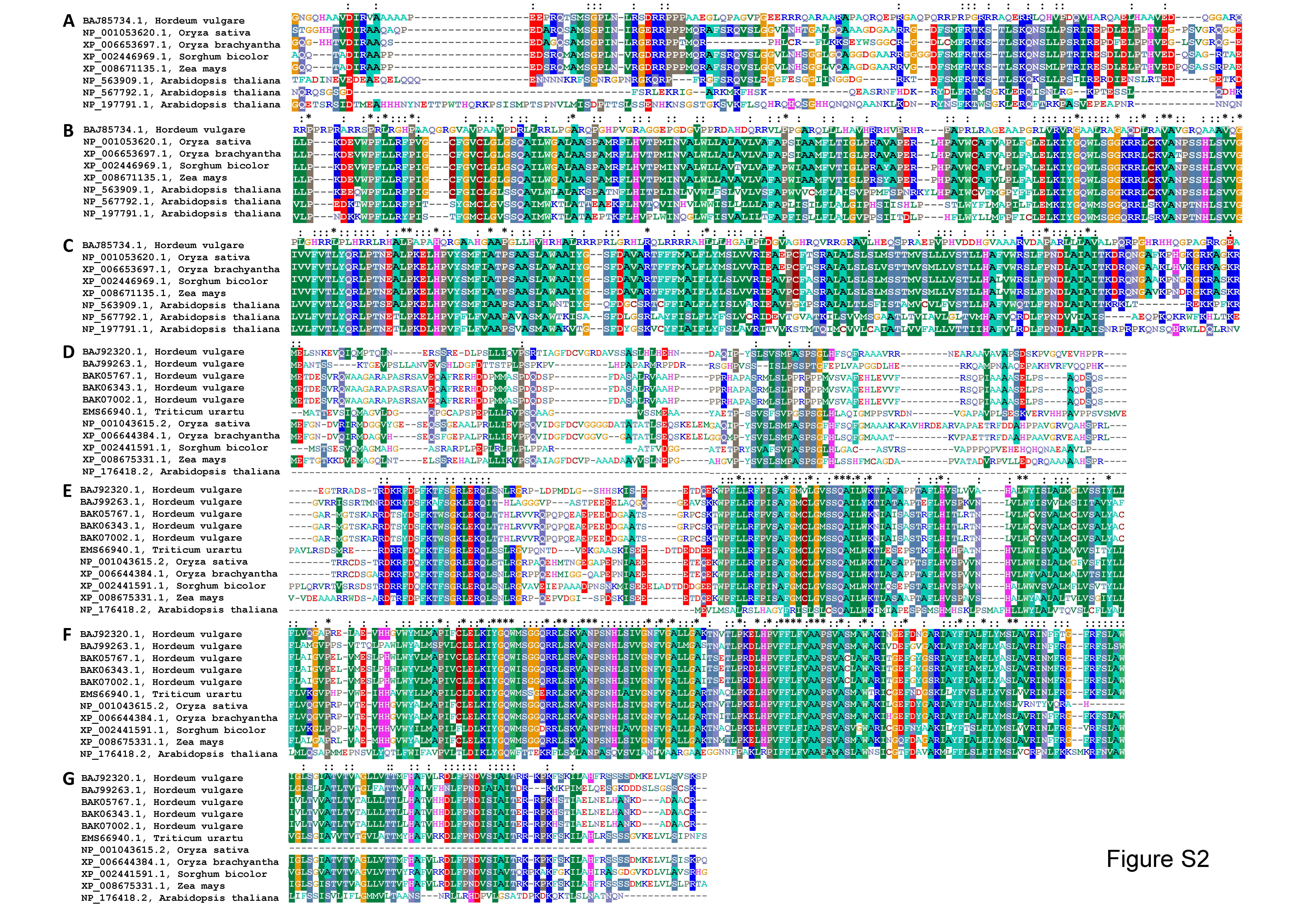

Supplement: Figure S2 — Alignment of HvSLAC1 and HvSLAH1 with homologous proteins in selected species. (A-C) Alignment of HvSLAC1 and some SLACs from monocot plant species and Arabidopsis. (D-G) Alignment of HvSLAH1 and some S-type anion channel SLAH from monocot plant species and Arabidopsis. Identical residues among the other proteins are indicated with asterisks. Colons indicate amino acids with high similarity among the proteins. Dashes indicate gaps. [file Image2.JPEG]
