# Supplementary material for: Linking stomatal traits and expression of slow anion channel genes HvSLAH1 and HvSLAC1 with grain yield for increasing salinity tolerance in barley
Source: Front Plant Sci. 2014 Nov 25;5:634. doi: 10.3389/fpls.2014.00634 (PMC4243495; doi:10.3389/fpls.2014.00634)
Supplement: Supplementary file 3 [file Table1.DOCX]

**Linking stomatal traits and expression of slow anion channel genes *HvSLAH1* and *HvSLAC1* with grain yield for increasing salinity tolerance in barley**

Xiaohui Liu^1,2,†^, Michelle Mak^1,†^, Mohammad Babla^1^, Feifei Wang^3^, Guang Chen^4^, Filip Veljanoski^1^, Gang Wang^5^, Sergey Shabala^3^, Meixue Zhou^3^, Zhong-Hua Chen^1,*^

^1^School of Science and Health, University of Western Sydney, Penrith, 2751, NSW, Australia

^2^School of Chemical Engineering and Technology, Tianjin University, Tianjin 300072, China

^3^School of Land and Food, University of Tasmania, Hobart, TAS 7005, Australia

^4^College of Agriculture and Biotechnology, Zhejiang University, Hangzhou, 310058, China

^5^School of Environmental Science and Engineering, Tianjin University, Tianjin 300072, China

***Correspondence:** Dr. Zhong-Hua Chen; Tel: +61 245701934; Fax: +61 245701383

Email: [Z.Chen@uws.edu.au](mailto:Z.Chen@uws.edu.au)

**^†^** These authors contributed equally for this work.

**Running title:** **Linking stomatal regulation to barley salinity tolerance**

**Table S1.** The primers used in RT-PCR and q-RT-PCR experiments.

| Gene | Forward Primer | Reverse Primer | Amplicon Size (bp) |
| --- | --- | --- | --- |
| *HvSLAC1* | TGGTGTCGCTGCTGCTC | ACCGCTTGATGTCGTTC | 165 |
| *HvSLAH1* | CGAGCACAACAACAGGGACA | TGACGGAAAGGGAGTAGGGTAT | 113 |
| *HvACTIN* | AATGGTCAAGGCTGGTTTCG | TCCTTCTGCCCCATCCCTAC | 108 |

**Table S2.** Correlation analysis between the expression of slow anion channel genes and grain yields in response to salinity in the Field Trial and Glasshouse Trial 3.

| **Parameters** | **GYFC** | **GYFT** | **RGYF** | **VSTS** | **GYGC** | **GYGT** | **RGYG** | **RICC** | **RICT** | **RRIC** | **RIHC** | **RIHT** | **RRIH** | **QRCC** | **QRCT** | **RQRC** | **QRHC** | **QRHT** | **RQRH** |
| --- | --- | --- | --- | --- | --- | --- | --- | --- | --- | --- | --- | --- | --- | --- | --- | --- | --- | --- | --- |
| **GYFC** | 1 |  |  |  |  |  |  |  |  |  |  |  |  |  |  |  |  |  |  |
| **GYFT** | 0.832** | 1 |  |  |  |  |  |  |  |  |  |  |  |  |  |  |  |  |  |
| **RGYF** | 0.163 | 0.672** | 1 |  |  |  |  |  |  |  |  |  |  |  |  |  |  |  |  |
| **VSTS** | 0.315 | 0.583* | 0.645** | 1 |  |  |  |  |  |  |  |  |  |  |  |  |  |  |  |
| **GYGC** | -0.446 | -0.401 | -0.164 | -0.045 | 1 |  |  |  |  |  |  |  |  |  |  |  |  |  |  |
| **GYGT** | -0.139 | -0.118 | -0.041 | -0.064 | 0.468 | 1 |  |  |  |  |  |  |  |  |  |  |  |  |  |
| **RGYG** | 0.178 | 0.411 | .508* | 0.148 | -0.566* | 0.148 | 1 |  |  |  |  |  |  |  |  |  |  |  |  |
| **RICC** | -0.376 | -0.520* | -0.430 | -0.617* | 0.073 | -0.095 | -0.166 | 1 |  |  |  |  |  |  |  |  |  |  |  |
| **RICT** | -0.287 | -0.063 | 0.260 | 0.084 | -0.139 | -0.414 | 0.219 | 0.395 | 1 |  |  |  |  |  |  |  |  |  |  |
| **RRIC** | 0.212 | 0.557* | 0.689** | 0.656** | -0.264 | -0.199 | 0.515* | -0.643** | 0.371 | 1 |  |  |  |  |  |  |  |  |  |
| **RIHC** | 0.070 | -0.202 | -0.434 | -0.461 | -0.405 | 0.148 | 0.284 | 0.304 | -0.060 | -0.391 | 1 |  |  |  |  |  |  |  |  |
| **RIHT** | 0.011 | 0.164 | 0.332 | 0.057 | -0.434 | -0.001 | 0.502* | -0.216 | 0.219 | 0.272 | 0.489 | 1 |  |  |  |  |  |  |  |
| **RRIH** | -0.096 | 0.298 | 0.684** | 0.549* | 0.074 | -0.038 | 0.094 | -0.625** | 0.145 | 0.653** | -0.473 | 0.462 | 1 |  |  |  |  |  |  |
| **QRCC** | 0.334 | 0.065 | -0.308 | -0.366 | -0.220 | -0.421 | -0.338 | 0.281 | -0.232 | -0.508* | 0.229 | -0.123 | -0.377 | 1 |  |  |  |  |  |
| **QRCT** | 0.247 | 0.573* | 0.659** | 0.600* | -0.006 | -0.270 | 0.066 | -0.383 | 0.376 | 0.584* | -0.357 | 0.350 | 0.656** | -0.019 | 1 |  |  |  |  |
| **RQRC** | -0.047 | 0.397 | 0.774** | 0.538* | -0.168 | -0.134 | 0.541* | -0.287 | 0.581* | 0.777** | -0.212 | 0.491 | 0.640** | -0.523* | .672** | 1 |  |  |  |
| **QRHC** | 0.100 | 0.203 | 0.186 | -0.192 | -0.454 | -0.151 | 0.452 | 0.105 | 0.052 | 0.068 | 0.396 | 0.451 | 0.055 | 0.368 | 0.227 | 0.213 | 1 |  |  |
| **QRHT** | 0.124 | 0.587* | 0.924** | 0.539* | -0.264 | -0.172 | 0.564* | -0.432 | 0.322 | 0.745** | -0.333 | 0.442 | 0.696** | -0.273 | 0.605* | 0.801** | 0.277 | 1 |  |
| **RQRH** | -0.179 | 0.202 | 0.678** | 0.471 | 0.146 | -0.118 | -0.038 | -0.396 | 0.099 | 0.404 | -0.606* | 0.094 | 0.708** | -0.320 | 0.319 | 0.455 | -0.326 | .659** | 1 |

**Note:** *P < 0.05 **P < 0.01. GYFC, grain yield in the control in the field; GYFT, grain yield in salt treatment in the field; RGYF, Relative grain yield in the field; VSTS, visual salt tolerance score in the glasshouse; GYGC, grain yield in the control in the glasshouse; GYGT, grain yield in salt treatment in the glasshouse; RGYG, relative grain yield in the glasshouse; RICC, RT-PCR gel intensity of *HvSLAC1* in the control; RICT, RT-PCR gel intensity of *HvSLAC1* in salt treatment; RRIC, relative RT-PCR gel intensity of *HvSLAC1*; RIHC, RT-PCR gel intensity of *HvSLAH1* in the control; RIHT, RT-PCR gel intensity of *HvSLAH1* in salt treatment; RRIH, relative RT-PCR gel intensity of *HvSLAH1*; QRCC, quantitative RT-PCR of *HvSLAC1* in the control; QRCT, quantitative RT-PCR of *HvSLAC1* in salt treatment; RQRC, relative quantitative RT-PCR of *HvSLAC1*; QRHC, quantitative RT-PCR of *HvSLAH1* in the control; QRHT, quantitative RT-PCR of *HvSLAH1* in salt treatment; RQRH, relative quantitative RT-PCR of *HvSLAH1*.

**Table S3.** Effect of salinity stress on selected stomatal traits of the double haploid (DH) lines in Glasshouse Trial 2.

|  | **Aperture width/Length** | | | | **Stomatal pore area, µm^2^** | | | | **Guard cell volume, µm^3^** | | | |
| --- | --- | --- | --- | --- | --- | --- | --- | --- | --- | --- | --- | --- |
|  | **DH line** | **Control** | **200 mM NaCl** | **%** | **DH line** | **Control** | **200 mM NaCl** | **%** | **DH line** | **Control** | **200 mM NaCl** | **%** |
| **Top five** | CG098 | 0.114±0.016 | 0.146±0.013 | 128.9 | CG104 | 42.1±1.0 | 23.6±2.5 | 56.1 | CG017 | 3601±236 | 3152±242 | 87.5 |
|  | CG005 | 0.119±0.010 | 0.121±0.010 | 101.8 | CG076 | 84.9±7.3 | 46.5±8.3 | 54.8 | CG021 | 3252±148 | 2602±222 | 80.0 |
|  | CG001 | 0.102±0.013 | 0.099±0.010 | 97.3 | CG048 | 55.4±5.8 | 26.8±6.4 | 48.3 | CG093 | 4273±508 | 3378±588 | 79.0 |
|  | CG046 | 0.153±0.022 | 0.138±0.009 | 90.2 | CG099 | 84.2±5.5 | 40.0±0.9 | 47.5 | CG048 | 3699±232 | 2834±296 | 76.6 |
|  | CG045 | 0.167±0.016 | 0.142±0.012 | 85.1 | CG107 | 56.8±4.9 | 26.9±4.4 | 47.4 | CG088 | 3997±269 | 2949±174 | 73.8 |
| **Bottom five** | CG026 | 0.200±0.012 | 0.051±0.005 | 25.5 | CG068 | 120.0±10.9 | 13.6±1.8 | 11.3 | CG011 | 3762±263 | 1128±85 | 30.0 |
|  | CG101 | 0.278±0.011 | 0.067±0.009 | 24.0 | CG097 | 94.9±10.2 | 7.9±0.6 | 8.3 | CG099 | 4124±257 | 1230±100 | 29.8 |
|  | CG049 | 0.235±0.028 | 0.055±0.004 | 23.4 | CG036 | 119.1±6.0 | 9.9±0.9 | 8.3 | CG103 | 5505±309 | 1513±194 | 27.5 |
|  | CG099 | 0.241±0.013 | 0.054±0.007 | 22.3 | CG070 | 122.0±8.3 | 9.1±1.2 | 7.5 | CG101 | 5153±435 | 1391±162 | 27.0 |
|  | CG090 | 0.247±0.014 | 0.054±0.005 | 22.0 | CG090 | 132.7±8.5 | 9.7±0.7 | 7.3 | CG036 | 5916±223 | 1415±92 | 23.9 |

Note: Data are the top-five best and bottom-five least performing DH line ranked according to their percentage (%) of the values of stomatal traits under 200 mM NaCl treatment as compared to the control.
